# Role of medical doctors in promoting and supporting sports participation by people with disabilities: an exploratory study of medical doctors’ knowledge, practices and perceptions

**DOI:** 10.1136/bmjsem-2025-002847

**Published:** 2026-03-09

**Authors:** Samitha Samanmalee Gowinnage, Jessica Hill, Iain Dutia, Emma Beckman, Gaj Panagoda, Sean Tweedy

**Affiliations:** 1 The School of Human Movement and Nutrition Sciences, The University of Queensland, Brisbane, Queensland, Australia; 2 School of Health and Rehabilitation Sciences, The University of Queensland, Saint Lucia, Queensland, Australia; 3 School of Allied Health, Australian Catholic University - Brisbane Campus, Banyo, Queensland, Australia; 4 Queensland Academy of Sport, Brisbane, Queensland, Australia; 5 Queensland Centre for Olympic and Paralympic Studies, The University of Queensland, Brisbane, Queensland, Australia; 6 Health and Wellbeing Centre for Research Innovation, The University of Queensland, Brisbane, Queensland, Australia

**Keywords:** Disability, Prevention, Physician, Health promotion, Para-Athletes

## Abstract

**Objectives:**

Despite the well-documented benefits of sports for individuals with disabilities, participation remains low, and disability-related medical complications are a major barrier. Medical doctors, as trusted sources of health information on physical activity with sustained patient contact, are well-positioned to manage these issues and promote safe participation in sports. However, limited evidence exists regarding their role. This study explored medical doctors’ knowledge, practices and perceptions in promoting and supporting sports participation among people with disabilities.

**Methods:**

An online survey collected quantitative and qualitative data from registered medical doctors internationally who provided direct general or specialised medical care to people with disabilities.

**Results:**

A total of 168 medical doctors from 16 countries participated. Most (91.1%) recognised the benefits of sports participation for people with disabilities and acknowledged their role in supporting participation (76.8%), while 69.7% identified medical complications as a barrier to participation. However, many reported low confidence in key areas: awareness of local disability sport organisations (53.6%); guiding patients (49.4%) and identifying relevant medical complications. Only 15.5% (of 58) had experience with pre-participation assessments. Limited awareness of disability sports, identified as a major barrier, and increasing awareness was the most frequently suggested strategy to enhance doctors’ involvement.

**Conclusion:**

Although medical doctors recognised the benefits of sports for people with disabilities and their role in promoting and supporting participation, many reported low confidence in their knowledge of disability sports and in identifying potential medical complications that may hinder sport participation. Development of methods and materials is required to provide doctors with the knowledge and confidence to effectively promote sport participation among people with disabilities.

**WHAT IS ALREADY KNOWN ON THIS TOPIC**

- Despite known benefits, sports participation among people with disabilities remains low due to a range of barriers, including disability-related medical complications.
- As trusted sources of health information on physical activity, medical doctors are well-positioned to minimise medical risks and promote safe, sustained sport participation among individuals with disabilities.

**WHAT THIS STUDY ADDS**

- This study highlighted that medical doctors acknowledge their important role in promoting sports participation among individuals with disabilities, an area not previously explored in depth.
- However, they reported low confidence in their knowledge of disability sports and in identifying potential medical complications that may hinder sport participation of people with disabilities.

**HOW THIS STUDY MIGHT AFFECT RESEARCH, PRACTICE OR POLICY**

- The findings highlight the need for culturally and economically appropriate strategies to improve doctors’ awareness of disability sports and their confidence in identifying medical risks that may hinder the participation of individuals with disabilities in sports.

## Background

 People with disabilities benefit significantly from regular physical activity (PA) participation, which reduces the risk of morbidity and mortality and improves quality of life.^1 2^ PA is defined as ‘any bodily movement produced by skeletal muscle that results in energy expenditure’.^1^ Sport is a form of PA defined as ‘a human activity involving physical exertion and skill as the primary focus of the activity, with elements of competition where rules and patterns of behaviour governing the activity exist formally through organisations and is generally recognised as a sport’.^3^

Disability sports often require specialised adaptive equipment, skills and coaching. These sports are either specifically designed for individuals with disabilities (eg, Goalball for people with vision impairment) or adapted to ensure inclusivity (eg, swimming with specific adaptations).^4^ Depending on individual preferences and goals, participation in disability sports may be either recreational or competitive. Organisations like the International Paralympic Committee (IPC), Invictus Games Foundation, Special Olympics Organisation and VIRTUS-International Sports Federation play a key role in promoting competitive sports for individuals with disabilities.^5^,^8^ The Paralympic Games are a major international event aligned with the Olympic Games.^5^ Para sports refer to sports in which athletes with disabilities compete under the IPC Athlete Classification Code and are governed by the IPC recognised International Federations. While often used interchangeably, the term ‘Para sport’ is distinct from ‘disability sport’, as over 80% of Australians with disabilities are not eligible for Para sport.^9^ The Invictus Games cater to injured or sick military personnel,^6^ Special Olympics offers inclusive sport opportunities for individuals with intellectual disabilities^7^ and VIRTUS provides elite-level competition for athletes with intellectual impairments.^8^

Sports participation is known to enhance self-satisfaction, self-confidence, social integration and independence for individuals with disabilities.^10^,^12^ Further, sport has the unique capability to transcend linguistic, cultural and social barriers, promoting inclusivity and engagement.^13^ Consequently, the United Nations has recommended sport as a means of promoting PA among people with disabilities.^13^ However, their participation still remains low due to various barriers.^14 15^ One significant barrier is a higher risk of health complications associated with disabilities.^15 16^ For instance, individuals with spinal cord injury often experience impaired thermoregulation and autonomic dysfunction, while those with cerebral palsy may face seizure risk.^15^,^17^ These complications not only limit safe engagement in sports but are common reasons for discontinuation,^18^ often requiring specialised medical attention.^16^

Medical doctors can play an important role in managing health risks and supporting the safe, sustainable sport participation for individuals with disabilities.^19^ Further, their direct and sustained contact with this population uniquely positioned them to promote sports benefits, address secondary conditions and motivate participation through education and support.^20 21^ As trusted sources of health information on sport and PA, they are well-placed to influence decision-making around participation in PA and sports.^22 23^ To effectively promote sports participation for individuals with disabilities, it is essential that medical doctors have basic awareness about disability sports and available sport opportunities. Although previous research has explored the role of health professionals in promoting PA among people with disabilities, there remains a notable gap in the literature regarding the specific contribution of medical doctors in promoting sports within this population.^24 25^ This study aimed to explore a medical doctors’ practices, knowledge and perceptions in promoting and supporting sports participation for individuals with disabilities.

## Methods

### Study design

An exploratory study design incorporating an online survey was used to collect both quantitative and qualitative data.

### Participants and recruitment

Participants included registered medical doctors who provided direct general or specialised medical care to people with disabilities and who might recommend sports or PA as a part of their role (eg, General practitioners, Rehabilitation physicians, Paediatricians, etc). To capture a broader spectrum of insights, we recruited participants from both high-income countries (HICs) and middle-income countries (MICs) as defined by the World Bank.^26^ The study was not designed to analyse differences between these groups, but the results are presented separately and in aggregate to provide an indication of areas where future approaches may require tailoring.

### Questionnaire development

Due to the lack of existing questionnaires, the questionnaire was purpose-designed based on an extensive review of literature^25 27^ and research team expertise (SSG, JH, ID, GP, EB, ST). The initial draft was piloted with 10 doctors in Australia and Sri Lanka to ensure clarity, flow, relevance and timing.^28^ Feedback was used to refine the questionnaire for face and content validity.^28^ The final 25-item tool included questions on demographics (9), knowledge and perceptions (7), and practices (9) in supporting disability sports participation, with both open and closed-response format. It required approximately 10–15 min to complete. The questionnaire can be found in online supplemental material 1.

### Patient and public involvement

Individuals with disabilities participating in the Para START project, run by our research group, helped shape the research focus, with some survey questions informed by their experiences with doctors. Para START is a performance-focused sports training programme tailored to participants’ interests and physical abilities, ensuring safe and effective participation. The programme is delivered by a multidisciplinary team, including exercise physiologists, a medical doctor, physiotherapist, occupational therapist, nutritionist and psychologist.^29^

### Data collection

The questionnaire was distributed through the online survey platform Qualtrics (20) allowing data to be collected while maintaining participants’ anonymity. The survey was configured to prevent multiple entries, and the link was distributed through professional networks and promoted on social media platforms (Facebook & WhatsApp). Snowball sampling was employed as participants were encouraged to share the survey link within their networks and as such, a response rate cannot be calculated. The survey was open from 1 July to 30 September 2024. Participants’ informed consent was obtained prior to commencing the online survey.

### Data analysis

Deidentified survey data were exported from Qualtrics into SPSS V.30, and missing data and ineligible responses were manually reviewed. Descriptive statistics were used for closed-response questions, and the frequencies of the responses were converted into percentages. Content analysis was used to analyse qualitative data derived from four open-ended questions on perceived advantages, disadvantages, actions taken to promote sports and views on supporting sport participation.^30^ Responses were read multiple times to familiarise the data and to identify common words and ideas. To enhance rigour, coding was conducted independently by two authors (SSG and JH) manually and the preliminary categories were identified. Frequency of each category was noted. Categories were refined and finalised through a consensus meeting involving all other researchers. Supportive example quotes were selected to provide contextual and better explanation of the findings.

### Equity, diversity and inclusion statement

This study sample included doctors from lower middle-income countries (LMICs), an under-represented group in disability sport research. The author team comprised three women and three men, five Australians and one Sri Lankan, with expertise in medicine, physiotherapy, exercise physiology and occupational therapy. One author has lived experience of disability and para sports. A commitment to equity, diversity and inclusion was maintained throughout the research process.

## Results

### Demographics, professional background and clinical practice of participants

A total of 176 medical doctors participated in the survey; data for 8 doctors were excluded from the analysis as they belonged to specialties that do not typically provide direct medical care to people with disabilities (eg, Anaesthesia, Pathology, Radiology, etc). Of the 168 included doctors, the majority were practising in Sri Lanka (n=77, 45.8%), Australia (n=56, 33.3%) and the UK (n=19, 11.3%). Participants represented HICs (n=83, 49.4%) and MICs (n=85, 50.6%). Of the 85 participants from MICs, 80 (94.7%) participants were from LMICs such as Sri Lanka, Bangladesh, Nepal and Bhutan. Participants’ practising countries are presented in table 1.

**Table 1:** Countries of participants’ current practice

Participants’ practising country	n (%)
High-income countries (83, 49.4%)	
Australia	56 (33.3)
UK of Great Britain and Northern Ireland	19 (11.3)
New Zealand	2 (1.2)
USA	1 (0.6)
Hong Kong (S.A.R.)	1 (0.6)
Singapore	1 (0.6)
Kuwait	1 (0.6)
UAE	1 (0.6)
Oman	1 (0.6)
Middle-income countries (85, 50.6%)	
Sri Lanka	77 (45.8)
Thailand	2 (1.2)
Indonesia	2 (1.2)
Bangladesh	1 (0.6)
Bhutan	1 (0.6)
Nepal	1 (0.6)
Serbia	1 (0.6)

Many (n=134, 79.8%) held postgraduate medical qualifications in addition to their foundational medical degrees, including postgraduate diplomas in medical specialities (n=8, 4.8%), master’s in medical specialities (n=9, 5.4%), Doctor of Medicine (MD) (n=48, 28.6%), Doctor of Philosophy (PhD) (n=1, 0.6%), as well as memberships and fellowships from medical colleges (n=68, 40.5%). Participant demographics and professional background details are presented in table 2.

**Table 2:** Demographics, professional and clinical practice of participants

Participants	High-income countries, n (%)	Middle-income countries, n (%)	Total, n (%)
	83	85	168
Postgraduate medical qualifications			
Yes	76 (91.6)	58 (68.2)	134 (79.8)
No	7 (8.4)	27 (31.8)	34 (20.2)
Specialist/training to be a specialist*	78 (94.0)	51 (60.0)	129 (76.8)
General practice	25 (30.1)	7 (8.2)	32 (19.0)
Rehabilitation	14 (16.9)	6 (7.1)	20 (11.9)
Psychiatry	7 (8.4)	7 (8.2)	14 (8.3)
General medicine	5 (6.0)	3 (3.5)	8 (4.8)
Paediatrics	4 (4.8)	4 (4.7)	8 (4.8)
Sport medicine	4 (4.8)	2 (2.3)	6 (3.6)
Emergency medicine	4 (4.8)	0	4 (2.4)
Neurology	0	2 (2.3)	2 (1.2)
Other†	15 (18.1)	20 (23.5)	35 (20.8)
Not a specialist/not in a specialist training pathway	5 (0.6)	34 (40.0)	39 (23.2)
General practice/primary care	0	5 (5.9)	5 (3.0)
Emergency medicine	1 (1.2)	5 (5.9)	6 (3.6)
General medicine	3 (3.6)	10 (11.8)	13 (7.7)
Nephrology	0	1 (1.2)	1 (0.6)
Neurology	0	2 (2.3)	2 (1.2)
Orthopaedic	0	3 (3.5)	3 (1.8)
Rheumatology	0	4 (4.7)	4 (2.4)
Rotational RMO	1 (1.2)	0	1 (0.6)
Oncology	0	1 (1.2)	1 (0.6)
Paediatric	0	3 (3.5)	3 (1.8)
Years of practice			
<8 years	11 (13.2)	30 (35.3)	41 (24.4)
8–16 years	50 (60.2)	45 (52.9)	95 (56.5)
More than 16 years	22 (26.5)	10 (11.8)	32 (19.1)
Type of workplace**			
Government hospital	58 (69.8)	72 (84.7)	130 (77.4)
Private hospital	9 (10.8)	6 (7.1)	15 (8.9)
Private practice	22 (26.5)	8 (9.4)	30 (17.9)
Community	7 (8.4)	2 (2.3)	9 (5.4)
Not-for-profit	1 (1.2)	3 (3.5)	4 (2.4)
Other‡	2 (2.4)	6 (7.1)	8 (4.8)
Workplace location**			
Metropolitan/urban/town/city	57 (68.7)	59 (69.4)	116 (69.0)
Rural/semi-urban/small city	26 (31.3)	24 (28.2)	50 (29.8)
Remote/isolated region	0	2 (2.4)	2 (1.2)
The types of disabilities encountered in practice**			
Physical disability (SCI, amputee, CP, etc)	54 (65.1)	54 (63.5)	108 (64.7)
Psychosocial (dementia, mental illness, etc)	54 (65.1)	33 (38.8)	86 (51.2)
Head injury/stroke/acquired brain injury	40 (48.2)	38 (44.7)	78 (46.4)
Intellectual disability (Down’s syndrome, Fragile X syndrome, etc)	39 (47.0)	25 (29.4)	64 (38.1)
Neurodiverse disability (ADHD, autism)	40 (48.2)	21 (24.7)	61 (36.3)
Sensory or speech disability (blindness, deafness, speech difficulties, etc)	31 (37.3)	27 (31.8)	58 (34.5)
Other§	1 (1.2)	2 (2.3)	3 (1.8)

* Participants were allowed to choose more than one answer.

† Cardiology, Community medicine, Endocrinology, Family medicine, Genitourinary medicine, geriatrics physician, nutrition, ophthalmology, rheumatology.

‡ Forensic hospitals and prisons, Government Institutions, Ministry of Health, Semi Government, Sporting Institute/National team, University Hospital.

§ Joint hypermobility /Ehlers Danlos, Neurological spasticity.

ADHD, attention-deficit hyperactivity disorder; CP, cerebral palsy; RMO, Resident Medical Officer; SCI, spinal cord injury.

A total of 129 (76.8%) doctors were either specialists or in specialist training at the time of data collection. Of these, 32 (19.0%) were General doctors, 20 (11.9%) were Rehabilitation physicians and 35 (20.8%) were from ‘Other specialities’ such as Cardiology, Community medicine, Endocrinology, Genitourinary medicine, Geriatrics, Nutrition, Ophthalmology and Rheumatology. Meanwhile, 39 (23.2%) doctors were not on a specialty pathway but were working in General medicine (n=13, 70.7%), Emergency medicine (n=6, 3.6%), General practice/primary care (n=5, 3.0%), etc.

Most participants (n=130, 77.4%) were employed in government hospitals, predominantly located in metropolitan areas (n=116, 69.0%) and had between 8 and 16 years of medical experience (n=95, 56.5%). Almost all participants (n=163, 97.0%) reported clinical experiences across a diverse range of disabilities.

### Medical doctors’ confidence in their knowledge of disability sports

Table 3 shows participants’ confidence in their knowledge of disability sports, assessed through three items: identifying medical complications, guiding patients to appropriate sporting organisations, and awareness of major disability organisations in their country. Confidence was rated on a five-point Likert scale (very confident to not at all confident).

**Table 3:** Medical doctors’ knowledge and perception of sports participation among people with disability

Participants	High-income countries, n (%)	Middle-income countries, n (%)	Total, n (%)
	83	85	168
*Medical doctors’ confidence in their knowledge of disability sports*			
Confidence in the ability to identify potential medical complications which could prevent sports participation of people with disabilities			
Not at all confident	7 (8.4)	6 (7.1)	13 (7.8)
Slightly confident	14 (16.9)	9 (10.6)	23 (13.7)
Neutral	19 (22.9)	23 (27.0)	42 (25.0)
Moderately confident	37 (44.6)	38 (44.7)	75 (44.6)
Very confident	6 (7.2)	8 (9.4)	14 (8.3)
Not responded	0	1 (1.2)	1 (0.6)
Confidence in the ability to guide a patient to the right sporting organisation for them			
Not at all confident	29 (34.9)	31 (36.5)	60 (35.7)
Slightly confident	11 (13.2)	12 (14.1)	23 (13.7)
Neutral	22 (26.5)	22 (25.9)	44 (26.2)
Moderately confident	14 (16.9)	16 (18.8)	30 (17.9)
Very confident	7 (8.4)	4 (4.7)	11 (6.5)
Confidence in awareness of major disability sport organisations in the country where currently practise			
Not at all confident	29 (34.9)	34 (40.0)	63 (37.5)
Slightly confident	13 (15.7)	14 (16.5)	27 (16.1)
Neutral	17 (20.5)	23 (27.1)	40 (23.8)
Moderately confident	22 (26.5)	10 (11.7)	32 (19.0)
Very confident	2 (2.4)	4 (4.7)	6 (3.6)
*Medical doctors’ perception of disability sports*			
The importance of medical doctors in promoting sports participation for people with disabilities			
Neutral	1 (1.2)	2 (2.3)	3 (1.8)
Moderately important	18 (21.7)	18 (20.4)	36 (21.4)
Very important	64 (77.1)	18 (20.4)	129 (76.8)
The medical complications serve as a barrier to sports participation for many people with disabilities			
Strongly disagree	4 (4.8)	1 (1.2)	5 (2.9)
Disagree	13 (15.7)	1 (1.2)	22 (13.1)
Neither agree nor disagree	11 (13.2)	13 (15.3)	24 (14.3)
Agree	45 (54.2)	46 (54.1)	91 (54.2)
Strongly agree	10 (12.1)	16 (18.8)	26 (15.5)

Approximately half (n=78, 46.5%) reported ‘neutral’ or ‘low confidence’ in their ability to identify medical complications potentially preventing individuals with disabilities from sports participation. Moreover, a substantial proportion of participants expressed low confidence in guiding patients to appropriate sports organisations (n=83, 49.4%) and in their awareness of major disability sporting organisations within their countries (n=90, 53.6%).

Within the open-response questions, participants reported several advantages and disadvantages of sport participation for people with disabilities. These are presented in table 4.

**Table 4:** Potential advantages and disadvantages of sports participation for people with disabilities

Categories	Example quotes
*Potential advantages (n=153 respondents)*	
Improves overall health and quality of life. n=129 (84.3%), HIC=65 (50.4%), MIC=64 (49.6%)	‘Increased muscle strength, improved balance and motor skills, enhanced heart health and higher energy levels.’ ‘I feel people with any disability are in stressful situations. To overcome that and distract them, they can use sports. At the same time, it releases natural endorphins.’
Self-confidence/self-esteem and self-satisfaction. n=44 (28.8%), HIC=24 (54.5%), MIC=20 (45.4%)	‘Boosting self-confidence and esteem.’
Social relationships and inclusion. n=36 (23.5%), HIC=27 (75.0%), MIC=9 (25.0%)	‘Providing a feeling of belonging to the community.’ ‘Improve social participation.’
Prevents disability-related complications and supports rehabilitation. n=13 (8.5%), HIC=4 (30.8%), MIC=9 (69.2%)	‘They can build up their muscle mass and strength to prevent mobility-insured sarcopenia, and they can prevent being obese and getting NCDs*.’*
Improves independence and function. n=11 (7.2%), HIC=10 (90.9%), MIC=1 (9.1%)	‘Enhances functional independence’
*Potential disadvantages (n=150 respondents)*	
Increased risk of injury. n=69 (46.0%), HIC=34 (49.3%), MIC=35 (50.7%)	‘Risk of sports-related injuries, eg, overuse injuries of upper extremities’
Increased risk of disability-related complications. n=28 (18.7%), HIC=15 (53.6%), MIC=13 (46.4%)	‘Possibility of getting medical complications during sports activities, e.g. Autonomic Dysreflexia and pressure sores in spinal cord injury’ ‘Some sports activities can be harmful to a particular disability; swimming can be harmful to TBI with seizures.’
The need for appropriate support and special facilities. n=14 (9.3%), HIC=9 (64.3%), MIC=5 (35.1%)	‘Someone has to assist them continuously and they need person-specified settings.’
Financial burden. n=13 (8.7%), HIC=9 (69.2%), MIC=4 (30.8%)	‘Increased expenses and difficulty in finding assistive devices compatible with sports.’
Negative impact on mental well-being. n=10 (6.7%), HIC=6 (60.0%), MIC=4 (40.0%)	‘Competitive sports could have a negative impact on their psychological well-being.’
No associated disadvantages. n=17 (11.3%), HIC=10 (58.8%), MIC=7 (41.2%)	‘I do not see any disadvantages’ ‘If the person involved in the right sport suitable for his disability, with adequate safety measures and proper guidance, I think there are no disadvantages’

HIC, high-income country; MIC, middle-income country; NCD, non communicable diseases.

#### Potential advantages

Five distinct categories were identified from the descriptions provided by 153 participants (91.1%). Most (n=129, 84.3%) emphasised the benefits of sport on the overall health and quality of life of individuals with disabilities, with improving mental well-being being identified more frequently than physical well-being. The second most cited advantage (n=44, 28.8%) was enhancing self-confidence/self-esteem and self-satisfaction. 36 participants (23.5%) noted improving social relationships and inclusion, while 13 (8.5%) rated preventing disability-related complications and supporting rehabilitation. Only 11 (7.2%) participants pointed out the contribution of disability sports to improved independence and functional capacity for individuals with disabilities.

#### Potential disadvantages

17 participants (11.3%) reported no associated disadvantages, with three of these participants specifically noting this absence of disadvantages when adequate safety measures and proper guidance were implemented. Among those who described potential disadvantages, five categories emerged. The most frequently reported concern (n=69, 46.0%) was an increased risk of injury. Additionally, 28 (18.7%) specifically cited the risk of disability-related medical complications, such as autonomic dysreflexia and pressure injuries in athletes with spinal cord injuries. Some also expressed concerns that sports could interfere with essential medical follow-ups, potentially worsening existing health conditions. A further 14 (9.3%) mentioned the need for appropriate support and special facilities, including personal assistance and accessible training facilities as disadvantages. 13 participants (8.7%) cited financial burdens, including costs for assistive devices and specialised facilities. Additionally, 10 (6.7%) noted the potential negative impact on mental well-being due to the stress and competitiveness of sports.

### Medical doctors’ perception of disability sports

Table 3 presents participants’ perceptions concerning the importance of medical doctors promoting sports participation among individuals with disabilities, alongside their views on the extent to which medical complications act as a barrier to such participation. Responses were rated on a five-point Likert scale. Most (n=129, 76.8%) perceived that the role of medical doctors in promoting sports participation for people with disabilities is ‘very important’. Additionally, many (n=117, 69.7%) either ‘agreed’ or ‘strongly agreed’ that medical complications serve as a barrier to sport participation for people with disabilities.

### Medical doctors’ current practices

Among the 88 (52.4%) medical doctors who had encountered patients with disabilities interested in sports, 65 (73.9%) reported supporting their participation. Among these, 58 (89.2%) described support methods, which grouped into seven categories (table 5). The most common was ‘Encouragement and emotional support’ (n=19, 32.7%) for sports participation. Others included facilitating connections with disability sports communities and organisations (n=17, 29.3%) and referred patients to the appropriate specialist (n=13, 22.4%) such as occupational therapists, social workers or exercise physiologists. Of nine (15.5%) medical doctors who conducted pre-participation medical evaluations, only five specified the screening tool used, such as Physical Activity Readiness Questionnaire (PARQ) and medical clearance forms issued by sport organisations.

**Table 5:** Medical doctors’ actions and suggestions for supporting people with disabilities to participate in sports

Categories	Example quotes
*What actions did medical doctors take to support their patients with disabilities in participating in sports?, n=58*	
Encouragement and emotional support. n=19 (32.7%), HIC=14 (73.7%), MIC=5 (26.3%)	‘Encouraged parents/ carer to seek guidance from the local council for sports facilities and training.’
Facilitated connections with disability sports communities and organisations. n=17 (29.3%), HIC=11 (53.8%), MIC=6 (46.1%)	‘Arranged coordination with National Paralympic committee’ ‘Make connect them with para-athletes’
Referred to the appropriate specialist. n=13 (22.4%), HIC=11 (84.6%), MIC=2 (15.4%)	‘Discussed and referred to Social worker, OT and Exercise physiologist. Explored with social support services.’
Managing medical conditions related to sports participation. n=10 (17.2%), HIC=7 (70.0%), MIC=3 (30.0%)	‘Treated focal neurological spasticity/muscle spasms with botulinum toxin.’ ‘Adjust the timing of medications with sedative effects.’
Educating them on safe options in sports participation. n=10 (17.2%), HIC=8 (80.0%), MIC=2 (20.0%)	‘Provide information, follow up with progress and educate about possible injuries.’
Pre-participation medical evaluation and n=9 (15.5%), HIC=6 (66.7%), MIC=3 (33.3%)	‘Provision of medical recommendations to continue.’
Providing of assistive equipment. n=2 (3.4%), HIC=2 (100%), MIC=0	‘Provide appropriate devices (e.g. recreational prosthesis for amputees) and equipment.’
*Medical doctors’ thoughts on supporting patients with disabilities to participate in sports or the potential of a screening tool to assist them with this process, n=48*	
Needs for further education and training of medical doctors regarding disability sports. n=8 (16.7%), HIC=6 (60.0%), MIC=2 (20.0%)	‘It can be done by a medical doctor at the point of contact but there should be awareness in medical practitioner which need to build up.’ ‘I think there should be more awareness regarding the need to promote sports/physical activity for patients with disability. Should be included in the Rehab training curriculum.’
The usefulness of a screening tool. n=9 (18.7%), HIC=6 (66.7%), MIC=3 (33.3%)	‘A screening tool will improve the practitioner’s confidence in decision-making and encourage more involvement with helping patients with disabilities to participate in sports.’
Enhancing the availability of accessible sporting opportunities. n=8 (16.7%), HIC=4 (50.0%), MIC=4 (50.0%)	‘The biggest barrier for participation is not medical issues, unavailability of facilities for them in my country.’
Raising societal awareness of disability sports benefits. n=5 (10.4%), HIC=3 (60.0%), MIC=2 (40.0%)	‘Suggest reaching out to the Children’s Hospitals and residential facilities and to educate, identify the needs and encourage people to involve in sports.’
Providing economic support. n=4 (8.3%), HIC=1 (25.0%), MIC=3 (75.0%)	‘These patients can be given additional financial assistance to encourage them to participate in sports.’
Other. n=6 (12.5%), HIC=3 (50.0%), MIC=3 (50.0%)	‘Effective counselling.’ ‘Acceptance of sport participation may be delayed for 6 to 12 months post-injury.’ ‘Conducting an audit for such patients and directing through the clinics will be beneficial.’

HIC, high-income country; MIC, middle-income country.

Of the 23 medical doctors who had never supported patients interested in disability sports, 20 (86.9%) provided reasons for their inaction. All 20 (100%) cited a lack of awareness about available disability sports as a primary reason. Other key reasons included lack of access to resources, facilities or programmes for disability sports in their practice area (n=19, 95.0%), concerns about potential health risks (n=7, 35.0%), lack of time due to busy clinical schedules (n=5, 25.0%) and perception that supporting sport participation falls outside their professional role (n=4, 20.0%).

### Medical doctors’ suggestions

48 (28.6%) participants provided recommendations to support patients with disabilities in sport or for the use of a screening tool to assist this process. Their recommendations were grouped into six categories. Eight participants (16.7%) emphasised the need for further education and training of medical doctors regarding disability sports. Nine participants (18.7%) recognised the usefulness of a screening tool to boost their clinical confidence. Others suggested enhancing the availability of accessible sporting opportunities (n=8, 16.6%), raising societal awareness of disability sports benefits (n=5, 10.4%) and providing economic support (n=4, 8.3%). Other recommendations (n=6, 12.5%) that did not fall under a specific theme included integrating disability sports referrals in clinical settings, the need for certified sports classifiers, providing counselling and delaying sports introduction until ensuring their psychological readiness after injury.

When considering overall responses for both open-ended and close-ended questions, the frequency and patterns of responses were largely consistent between medical doctors from HICs and MICs.

## Discussion

Promoting sport for individuals with disabilities requires general knowledge about disability sports and available sport providers. Although the role of health professionals in promoting PA for people with disabilities has been studied, this is the first known study to focus specifically on medical doctors’ knowledge, practices and perceptions in promoting sport participation among people with disabilities.^24 25^ The participants in this study also acknowledged the well-documented benefits of sport participation, including improving physical and mental health as well as quality of life.^10 11^ Further, they also recognised the role of medical doctors in promoting and supporting sports participation, consistent with prior research highlighting healthcare providers’ support for PA among patients with disabilities.^25 27 31^

Despite the positive perception of their role, participants had low confidence in their knowledge of disability sports and available local opportunities. This aligns with evidence that limited knowledge among medical doctors hinders sport promotion for people with disabilities.^24^ It is also consistent with evidence from Switzerland demonstrating that greater awareness of disability sports among healthcare professionals positively influenced their promotion of sports and PA for people with disabilities.^32^ As the preferred and trusted source of information on health and PA, enhancing doctors’ awareness in this area is important.^22 23^ Participants also identified this knowledge gap as a major barrier to their involvement and suggested that addressing it should be the primary strategy to strengthen their capacity to promote sports participation.

In relation to the role medical doctors can play in supporting patients to participate in sport, most acknowledged that medical complications often hindered sports participation among people with disabilities. Disability-related medical complications are well-documented barriers to sport participation for individuals with disability^14 15^ and can be worsened by specific sports activities.^33^ For example, individuals with spinal cord injuries are at an increased risk of pressure sores due to sensory impairment which can be worsened by wheelchair sports.^33^ Further, a qualitative study also identified health issues coupled with inappropriate medical advice as a primary reason for discontinuation of sports participation among non-elite Para-athletes.^18^ Nonetheless, with adequate pre-participation evaluations and proper management and safety measures addressing these potential medical complications, individuals with disabilities can safely engage in sports and gain its benefits.^16 19 34^

While participants acknowledged medical complications as a significant barrier to sport participation for people with disabilities, nearly half reported lack of confidence in identifying these potential complications. Individuals with disabilities are frequent users of healthcare services; therefore, medical doctors are uniquely positioned to address these health complications and facilitate safe sports participation.^21^ However, previous research has highlighted a persistent gap in medical doctors’ knowledge regarding disability-specific health issues.^35 36^ For example, a systematic review revealed that GPs’ often have limited knowledge and expertise in managing disability-related health conditions largely due to insufficient exposure during medical education or residency training.^35 37^ However, the current study did not investigate the depth or specificity of medical doctors’ knowledge regarding the risk of medical complications that may hinder sport participation among individuals with disabilities. Therefore, further research is warranted to explore the extent and adequacy of their knowledge in this area.

In relation to how medical doctors conduct screenings and assessments for patients with disability who want to participate in sport, only five participants reported using screening tools, such as the PARQ or bespoke medical clearance forms from particular sports organisations. Currently, there are no universally accepted screening guidelines tailored to athletes with diverse impairments.^38^ Consequently, adapted versions of pre-participation tools originally designed for able-bodied athletes are commonly used for athletes with disabilities.^39^ Clearer guidance would help doctors better identify risks and ensure safer participation.

This study included medical doctors from both HICs and MICs to capture diverse perspectives on disability sports. Despite the economic context, medical doctors showed similar practices, knowledge and perceptions regarding disability sports, strengthening the robustness of the findings. Our findings suggest that HIC doctors were more likely to identify ‘social relationships and inclusion’ as a benefit of sport. However, as this study was not designed to examine cross-country differences, these observations should be interpreted cautiously. Future research should explore such cultural and contextual influences in greater depth.

To our knowledge, this is the first study to explore medical doctors’ knowledge, practices and perceptions in promoting sport participation among individuals with disabilities. A strength of this study is the inclusion of a substantial number of participants from LMICs, an underrepresented group in existing research on disability sports, helping to address an important gap in a field largely focused on HICs. Furthermore, the integration of both quantitative and qualitative data provides a more comprehensive understanding of the findings. Additionally, the involvement of medical doctors from various specialties provides a broader perspective on medical doctors’ role in promoting sport participation for people with disabilities.

### Limitations

While the use of an online survey facilitated data from doctors across various regions, including both HICs and MICs, interpreting study findings does require consideration of certain limitations. First, the sampling approach may have introduced selection bias, as those interested in disability sports were more likely to respond. Additionally, non-respondents or individuals who did not complete the survey may differ systematically from respondents, potentially affecting representativeness. Furthermore, social desirability bias may have affected responses, with participants potentially reflecting their practice and perceptions more positively than they truly are. Additionally, the use of Likert scales depends on participants’ self-reported, subjective interpretations, potentially affecting consistency. Future qualitative research could offer deeper, more nuanced insights.

### Clinical implications

This study revealed that medical doctors acknowledge their role in promoting and supporting sports participation among people with disabilities, a perspective not widely reflected in existing literature. The findings highlight the need for culturally and economically appropriate strategies to improve doctors’ awareness of disability sports and their confidence in identifying potential medical complications associated with participation, such as autonomic dysreflexia, thermoregulation challenges and pressure ulcers in individuals with spinal cord injuries. Furthermore, developing a comprehensive mapping of national and international disability sport organisations could assist doctors in guiding patients toward relevant resources and opportunities.

## Conclusion

This study highlighted the role of medical doctors in promoting and supporting sports participation among individuals with disabilities. While participants acknowledged the benefits of sports for people with disabilities as well as medical doctors’ role in promoting sport participation, the findings highlight a lack of confidence in their knowledge of disability sports and ability to identify medical issues that may hinder sports participation of people with disabilities. Furthermore, doctors reported barriers for their involvement and proposed strategies to enhance sport participation among individuals with disabilities.

## Supplementary material

10.1136/bmjsem-2025-002847 online supplemental file 1

## Data Availability

Data are available upon reasonable request.
