# Supplementary material for: Role of medical doctors in promoting and supporting sports participation by people with disabilities: an exploratory study of medical doctors’ knowledge, practices and perceptions
Source: BMJ Open Sport Exerc Med. 2026 Mar 9;12(1):e002847. doi: 10.1136/bmjsem-2025-002847 (PMC12983821; doi:10.1136/bmjsem-2025-002847)
Supplement: online supplemental file 1 [file bmjsem-12-1-s001.docx]

Supplemental material 1

**Research Title: The role of medical doctors in promoting and supporting sports participation by people with disabilities: Practices, knowledge and perceptions of medical doctors**

***Demographic information***

| 1. **Are you a registered medical practitioner** |
| --- |
| - Yes, I am a registered medical practitioner - No, I am not a registered medical practitioner |
| 1. **In which country/countries are you registered?..................................................................** |
|  |
| 1. **What medical qualifications do you have and in which country did you obtain them?** |
| - MBBS-……………………………. - MD-……………………………… - Other-………………………… |
| 1. **If you are a specialist/training to be a specialist doctor, what is your speciality? (please select all that apply)** |
| - General Practice - Medical rehabilitation - Community rehabilitation - Neurology - Paediatrics - Orthopaedics - Sports Medicine - other……………………………. |
| 1. **In which country do you currently practice?...................................................................** |
| 1. **How long have you been working within your medical profession?** |
| - < 8 years - 8 -16 years - >16 y |
| 1. **Where do you currently work (Please select all that applies)** |
| - Government Hospital - Private Hospital - Private Practice - Community - Not-for-profit - Other (please state)……………………………….. |
| 1. **In what location do you currently practice?** |
| - Metropolitan/Urban/Town/Major City - Rural/ Semi-urban/ small city - Remote/isolated region |
| 1. **What are the main diagnostic groups of your patients with disabilities (please select all that apply)**   **(Disability is an umbrella term for impairments of body function or structure, activity limitations or participation restrictions (WHO, ICF)** |
| - Physical disability (SCI, Amputee, CP, etc.) - Intellectual disability (Downs’ syndrome, Fragile X syndrome. Etc.) - Sensory or speech disability (blindness, deafness, speech difficulties, etc.) - Head injury/stroke/acquired brain injury - Psychosocial (dementia, mental illness etc.) - Neurodiverse disability (ADHD, Autism) - Chronic pain - other…………………………………….. |

***Knowledge and Perception of sport for people with disabilities***

| 1. **How important do you think it is that medical doctors promote sports participation for people with disabilities?** |
| --- |
| - Very important - Moderately important - Neutral - Slightly important - Not at all important |
| 1. **There are many different sports organisations, such as the Special Olympics, Paralympics, Invictus, etc.**   **How confident are you in your ability to guide a patient to the right sporting organisation for them?** |
| - Very confident - Moderately confident. - Neutral - Slightly confident - Not at all confident |
| 1. **In the boxes below, please indicate the extent to which you agree with the following statement: The potential for medical complications serves is a concern that prevents many people with disabilities from participating in sports?** |
| - Strongly agree - Agree - Neutral - Disagree - Strongly Disagree |
| 1. How confident are you in your ability to identify potential medical complications which could prevent sport participation of people with disabilities? |
| - Very confident - Moderately confident. - Neutral - Slightly confident - Not at all confident |
| \| 1. **How confident are you about your awareness of the main disability sport organizations in Australia/ in the country where you currently practice?** \| \| --- \| \| - Very confident - Moderately confident. - Neutral - Slightly confident - Not at all confident \|  1. **As a medical practitioner, what, if any, do you think are the potential advantages of sports participation for people with disabilities?.......................................................................................................................**   **…………………………………………………………………………………………………………………………………..** |
| 1. **As a medical practitioner, what, if any, do you think are the potential disadvantages of sports participation for people with disabilities? ………………………………………………………………………………. ..................................................................................................................................................** |

***Practice specific questions***

| 1. **In your clinical practice, have you seen patients who participate or want to participate in disability sports?** |
| --- |
| - Yes (go to Q.17) - No (go to Q.21) |
| 1. **Approximately, how many patients have you seen who participate or want to participate in disability sport?** |
| - < 10 - 10-20 - > 20 |
| 1. **For those who wanted to participate but were not currently, did you support their participation?** |
| - Yes (go to Q.19) - No (go to Q.20) |
| 1. **What did you do as a medical professional to support their participation?**   **…………………………………………………………………………………………………………………………………………………………………………………………………………………………………………………………………………………………………..** |
| 1. **Why didn't you support them to participate in sports? (Please select all apply)** |
| - Lack of my awareness about sports for people with disability - Lack of access to resources, facilities, or programs for sport participation of individuals with disabilities in my area. - I worry about the potential risks of injury or exacerbating existing medical conditions of the person through sports participation. - It is not my duty. - Due to my busy schedule in clinics, I do not have time for that. - Other ………………………………………………………………………………….. |
| 1. **Would you promote participation in sports for young patients with disabilities who express interest in sports when they come to you? (this Q applicable only, if the answer is “no” for Q 16)** |
| - Yes (go to Q. 23) - No (go to Q.22) |
| 1. **Why wouldn’t you promote participation in sports for young patients with disabilities who come to you? (Please select all that apply)** |
| - Lack of my awareness about sports for people with disability - Lack of access to resources, facilities, or programs for sport participation of individuals with disabilities in my area. - I worry about the potential risks of injury or exacerbating existing medical conditions of the person through sports participation. - It is not my duty. - Due to my busy schedule in clinics, I do not have time for that. - Other (please state) |
|  |
| 1. **Are there screening tools you use or are aware of that would be useful for assessing the risks associated with sports participation for different patient groups?** |
| - Yes (please specify)……………………………………………. - No |
| 1. **Do you have any additional thoughts on supporting patients with disabilities to participate in sport or the potential of a screening tool to assist you with this process?** |
| ………………………………………………………………………………………………………………………………………………………  ……………………………………………………………………………………………………………………………………………………… |
